# A Novel Dairy–Beetroot Powder: Microencapsulation Improves Stability and Sensory Qualities While Preserving Cardioprotective Bioactives

**DOI:** 10.3390/foods15081351

**Published:** 2026-04-13

**Authors:** Lucileno Rodrigues da Trindade, Diego dos Santos Baião, Davi Vieira Teixeira da Silva, Fernanda Petzold Pauli, Vania Margaret Flosi Paschoalin

**Affiliations:** 1 Department of Biochemistry, Chemistry Institute, Federal University of Rio de Janeiro (UFRJ), Avenida Athos da Silveira Ramos 149, Room 545, Cidade Universitária, Rio de Janeiro 21941-909, RJ, Brazil; lucileno.trindade@gmail.com (L.R.d.T.); diegobaiao20@hotmail.com (D.d.S.B.); davivieiraufrj@gmail.com (D.V.T.d.S.); 2 Graduate Studies in Food Sciences, Federal University of Rio de Janeiro (UFRJ), Avenida Athos da Silveira Ramos 149, Room 545, Cidade Universitária, Rio de Janeiro 21941-909, RJ, Brazil; 3 Department of Organic Chemistry, Chemistry Institute, Rio de Janeiro State University (UERJ), Rua São Francisco Xavier 524, Room 406, Maracanã, Rio de Janeiro 20550-900, RJ, Brazil; fernanda_pauli@hotmail.com

**Keywords:** beetroot, probiotic formulation, dietary nitrate, betacyanins, polysaccharide microcapsules, innovative powdered, beet–dairy beverage

## Abstract

Background: Beets are enriched in bioactive compounds with beneficial effects on cardiovascular function. Nitrate is a precursor for nitric oxide synthesis, exhibiting an effect on cardiomyocytes and myocardial ischemia/reperfusion, improving endothelial function and reducing arterial stiffness. Betanin, saponins and phenolic compounds, other beet compounds, can limit the generation of reactive oxygen species and modulate gene expression. However, it has been a challenge to develop beetroot formulations for the oral administration of these compounds while preserving pleasant sensory characteristics. Objective: The objective of this study was to develop an innovative dairy–beetroot powder drink, microencapsulated in polysaccharides, i.e., maltodextrin, cassava starch or a combination of both, that could be easily reconstituted. Key Results: The microencapsulated formulation following freeze-drying displayed low water activity (<0.30) and high solubility (>90%), with rapid dispersion in aqueous medium. Fourier transform infrared spectroscopy confirmed the preservation of functional groups from the dairy base and sugar beetroots. Thermogravimetry analyses pointed out a slight increase in thermal stability for the powder formulation. The microencapsulation efficiency of betalains reached 81% in the powder formulation that combined cassava starch and maltodextrin as encapsulation agents. The novel dairy–beetroot powder drink can be stored at room temperature, ensuring microbiological safety and preserving good sensory acceptance. Conclusions: Dairy–beetroot powder microcapsules emerge as an efficient food strategy to provide bioaccessible dietary nitrate and antioxidant compounds, overcoming flavor and stability limitations but still aiding in terms of its vascular and hemodynamic-protective effects.

## 1. Introduction

Beets (*Beta vulgaris* L.), belonging to the Chenopodiaceae family, stand out as a source of bioactive compounds such as nitrate, phenolic derivatives, organic acids, saponins, and betalains, which are associated with cardioprotective, antimicrobial, antiallergic, and anti-inflammatory benefits for human beings [1,2,3,4]. Furthermore, beetroot is considered a good source of dietary fiber and a wide range of essential minerals, such as potassium, sodium, iron, copper, magnesium, calcium, phosphorus, and zinc, as well as B-complex vitamins, besides retinol and ascorbic acid. Nevertheless, beetroot has high contents of dietary nitrate (NO_3_^-^), which, after being ingested, is absorbed by the jejunum and into the bloodstream or tissues, where it accumulates intracellularly. About 60% of the absorbed dietary NO_3_^-^ is excreted in urine, and 25% is transported to the salivary glands, being concentrated in saliva. In the oral cavity, NO_3_^-^ is converted into nitrite (NO_2_^-^) by NO_3_^-^-reductase. Subsequently, when it reaches the acid environment of the stomach, NO_2_^-^ is protonated, forming nitrous acid (HNO_2_), which spontaneously decomposes into nitric oxide (NO) and other bioactive nitrogen oxides [5,6,7].

NO is a 5–10 s half-life gaseous substance, with low molecular weight (−30.01 g·mol^-^) and a moderate water solubility of 1.94 mM atm^−1^ at 25 °C but augmented solubility in polar solvents and biological fluids [8,9]. NO regulates vascular tonus by interacting with the iron from the prosthetic heme group of soluble guanylate cyclase enzymes (GCs), forming cyclic guanosine monophosphate (cGMP), which promotes the relaxation of the adjacent smooth muscle cells. NO also promotes cytotoxic and cytostatic effects, causing the knock-down of microorganisms, parasites and even tumoral cells in a synergic response to the immune system. NO can also act as a neurotransmitter in the central and peripheral nervous systems, facilitating the release of other neurotransmitters and hormones. Other studies have reported NO effects on circulating monocytes and platelets for the maintenance of vascular homeostasis and control of smooth muscle cell proliferation and growth, as well as on the activation and aggregation of platelets, leukocytes and adhesion molecules during the inflammatory process [5,9,10]. Clinical trials have demonstrated that the intake of NO_3_^-^-enriched foods, in particular beetroots, can improve biochemical, hemodynamic and vascular parameters in healthy individuals and especially in those populations at risk of cardiovascular diseases (CVDs) [3,11,12,13,14,15].

However, consumption of fresh beetroot juice does not provide the effective concentration of NO_3_^-^ required for achieving beneficial endothelial and vascular effects. Furthermore, clinical tests have concluded that large beetroot juice volumes, 500–700 mL, the most common presentation of beets, besides containing lower than the pharmacological concentrations of NO_3_^-^—varying from 4.5 to 8 mmol (280–500 mg of NO_3_^-^)—have impaired the adherence of subjects to acute and long-term interventions [3,11,16,17,18]. Consumption of large volumes of beetroot juice can be uncomfortable, potentially causing early satiety, nausea, and gastrointestinal effects such as abdominal distension and digestive discomfort. Preparing and consuming large quantities of beetroot juice daily would require time and discipline, which does not fit well into most people’s routines [16,17,18]. Furthermore, low consumption of vegetables reflects a scenario where financial and logistical convenience overrides nutritional health. In terms of energy density, ultra-processed foods that are richer in flour, sugar, and fat are usually cheaper and have a longer shelf life compared with fresh vegetables. The low consumption of vegetables is aggravated by time availability, a scarce resource today, which makes it difficult to carry out preparation steps such as cleaning, washing, peeling, and cooking vegetables. Furthermore, vegetable consumption has been associated with restrictive diets, and this psychological burden leads many people to see vegetable consumption as an obligation, not pleasure [19]. Thus, the challenge remains in developing novel formulations that reconcile high NO_3_^-^ contents and concentrations of bioactive compounds, which have already been demonstrated to be very effective in terms of nutritional and cardiac indexes while still presenting attractive sensorial characteristics, practicality and consumer acceptance, allowing for adherence to nutritional intervention.

The food industry has developed more versatile and highly practical products for easy consumption. Among them, the elaboration of dairy beverages is an important and growing segment in the food sector in Brazil, reaching big populations, due to their recognized and inherent nutritional and health relevance [20]. Among the current technologies employed for food processing, microencapsulation emerges as an alternative to improve the storage, stability, and bioavailability of bioactive compounds, in addition to contributing to masking the unpleasant and astringent taste of phenolic compounds [21]. Microencapsulation involves the entrapment of a single or multiple active substances within a continuous, microscopic film of core encapsulating polymer material. Furthermore, the freeze-drying technique for microencapsulation has been used to encapsulate bioactive compounds from many foods. But the effect of beetroot processing on bioactive compounds, such as dietary NO_3_^-^, antioxidant compounds, and flavors, has not yet been evaluated [22].

Several polysaccharides derived from food plants and agro-industrial residues and recognized as safe (GRAS) by the United States Food and Drug Administration (FDA) can be employed to build microcapsules to load bioactive substances and present biodegradability, biocompatibility, and lower propensity to adverse effects [23]. Those food-grade polysaccharides—starch, maltodextrin, pectin, gum Arabic, xanthan gum, sodium alginate and others—are widely used as microencapsulating agents for bioactive food compounds, protecting them and facilitating their delivery under physiological conditions [24].

The present study has the purpose of developing a dairy–beetroot powder beverage by microencapsulating some functional beetroot compounds through freeze-drying with encapsulating agents, thus preserving the appropriate nutritional characteristics in polysaccharide microcapsules that protect and increase the core compound’s bioaccessibility and merging the benefits of beetroot compounds and the probiotic characteristics of a dairy beverage fermented by kefir microbiota. The yield of microencapsulated formulations and their characteristics, such as centesimal composition, microparticle size and morphology, and several physicochemical characteristics such as water activity and solubility, were determined with thermogravimetry and infrared absorption spectroscopy analyses. Contents of NO_3_^-^, NO_2_^-^ and betalains were estimated, and the sensory analyses of the beetroot dairy beverage and their acceptance were performed; further, the commercial shelf life of the microencapsulated formulations was determined through microbiological analysis by storing them for 30 days at room temperature.

## 2. Materials and Methods

### 2.1. Reagents

All reagents and standard solutions were purchased from Sigma-Aldrich Chemical Co. (St. Louis, MO, USA). High-performance liquid chromatograph (HPLC)-grade solvents, including methanol (MeOH), ethanol, and acetonitrile, were purchased from Tedia Company Inc. (Rio de Janeiro, RJ, Brazil). Inductively coupled plasma mass spectrometry (ICP-MS)-grade solvent, including nitric acid was purchased from Vetec Solutions (Rio de Janeiro, RJ, Brazil), and it was purified by sub-boiling and distilling git in a quartz still (Kürner Analysentechnik, Rosenheim, BY, Germany). The Merck IV multi-elemental standard solution (Merck, SP, Brazil) containing 29 elements in nitric acid was used to prepare the standard analytical curves. To evaluate the accuracy of the method, two reference materials, Certified Skimmed Milk Powder (ERM-BD150^®^) and Milk Powder 1549 (Sigma-Aldrich Chemical Co., Bellefonte, PA, USA), were employed. Regarding the culture media, yeast agar, glucose extract, chloramphenicol (YGC) and glucose agar were purchased from Hexis Científica (Campinas, SP, BRA), while Man, Rogosa and Sharpe (MRS) Agar was obtained from Acumedia (Lansing, MI, USA). The 3M Petrifilm™ Salmonella Express Sy and 3M Petrifilm™ *E. coli* Count Plate test systems were purchased from 3M Health Care (Campinas, SP, Brazil). Finally, all reagents were prepared with ultrapure water (resistivity > 18.2 MΩcm), obtained from a Milli-Q^®^ INTEGRAL 10 system (Millipore, MA, USA). All reagents and standard solutions used were of analytical grade.

### 2.2. Preparation of Beetroot Dairy Drinks

According to Normative Instruction No. 16 of 23 August 2005, from the Brazilian Ministry of Agriculture, Livestock and Supply (MAPA) [25], a dairy drink is defined as a beverage resulting from the mixture of milk, at different concentrations, and whey—liquid, concentrated or powdered—or other product(s) or another food substance(s), such as vegetable fat, fermented milk(s), selected lactic ferments and other dairy products. Furthermore, the dairy base must represent at least 51% (*w*/*w*) of the total ingredients of the final product and may or may not be flavored. Therefore, the dairy base was formulated using whole ultra-high-temperature (UHT) milk, whey powder and eXact^®^ Kefir 1 cultures (Novonesis, São Paulo, SP, Brazil) for fermentation for 6 h at 43 °C, considering the minimum concentration of 51% (*w*/*w*) of these ingredients in the final product formulation [25]. Beetroots (*Beta vulgaris* L.) purchased at local markets at Rio de Janeiro city, RJ, Brazil, with no signs of deterioration (cracks, stains or wet areas), were sanitized according to the Brazilian Health Regulatory Agency—ANVISA [26]. Afterwards, beetroots were cut into cubes, steamed at 100 °C and crushed in a food processor (Philips Walita-RI1836, São Paulo, SP, Brazil).

### 2.3. Preparation of Microencapsulated Beetroot Dairy Drinks

The microparticles were elaborated as described in [27] with some adaptations. The microencapsulation of the beetroot dairy beverage, with each encapsulating polysaccharide or combination of them, was performed considering the total solids in beetroot and dairy beverage. Each encapsulating polysaccharide, maltodextrin (M) or cassava starch (A) was mixed with the beetroot dairy beverage through stirring and homogenization using a GO stirrer-MS-H-Pro (São Paulo, SP, Brazil) at 600 rpm for 30 min followed by lyophilization (Terroni LS6000, São Paulo, SP, Brazil), and the sublimation of the aqueous samples was performed for 5 days at −55 °C under a vacuum pressure of 60–100 μHg, until the beverages reached a powdery appearance (dry). Three different formulations were produced, keeping the ratio of 2:1:1: (1) dairy base: beetroot: maltodextrin; (2) dairy base: beetroot: cassava starch; and (3) dairy base: beetroot: maltodextrin (50%) and cassava starch (50%). A fourth formulation without encapsulating agent (control) in the ratio of 2:1 was produced in the same way as (4) dairy base: beetroot. The microencapsulated beetroot dairy beverages were ground using a mortar and pestle, and the microparticles were packed in a sealed plastic container and maintained in a cool, dry, and dark place at room temperature, considering the necessary headspace to avoid product overflow during the sealing process.

### 2.4. Morphology, Physicochemical and Thermal Properties of Microcapsules

The morphology of microparticles was evaluated by scanning electron microscopy (SEM). Samples were covered with a thin layer of gold in a Balzers metallizer, Union FL 9496, and were deposited on double-sided carbon adhesive tape, fixed on the surface of the microscope base JEOL JSM 5310 (Jeol Ltd., Tokyo, Japan) at 15 kV.

The size distribution of the microparticles was determined by the laser diffraction technique by using a Mastersizer MicroPlus MAF 2000 analyzer (Malvern Panalytical Ltd., Grovewood Road, Malvern, WO, UK) from 0.02 to 2,000,000 μm. Samples were dispersed in water, reaching a 10% obstruction index after three consecutive readings, and the results were expressed as d (0.1), d (0.5) and d (0.9), corresponding to the maximum size, in μm, of 10%, 50% and 90% of the particles analyzed, in addition to the determination of the dispersion index “*span*” [28].

Infrared absorption spectra were obtained using a Fourier transform infrared spectrometer coupled to the attenuated total reflectance technique with ZnSe crystal (PerkinElmer Inc., Waltham, MA, USA). The analysis in the infrared region was 8300–350 cm^−1^ in transmittance mode, where twenty readings were collected and averaged with a resolution of 0.5 cm^−1^ and a signal-to-noise ratio of 10,000/1 pk-pk in 5 s of scanning [29].

The yield was calculated from the ratio between the mass of microparticles obtained after freeze-drying and the total mass of each sample, according to Equation (1):

Yield (%) = (formed microparticles(g) × 100)/(emulsion(g))(1)

The solubility index of microencapsulated beetroot dairy beverages was determined by adding 1 g of each sample to 100 mL of Milli-Q water, followed by homogenization for 5 min at 15,000 rpm and centrifugation at 3000× *g* for 5 min. Then, a supernatant aliquot was dried in an oven at 105 °C for 5 h, and the solubility (%) of each sample was calculated from the mass differences before and after the procedure [28], according to Equation (2):

Solubility index (%) = (m _dissolved(g)_/m _initial sample(g)_) × 100 (2)

Water absorption indexes were calculated as described in [30]: The powder samples were placed in a pre-weighed centrifuge tube and subsequently hydrated with deionized distilled water. The tube containing the sample and water was closed and shaken for a few seconds. The suspension rested for a specified period, with occasional shaking to maintain uniform hydration. The tube was centrifuged at 2000× *g* for exactly 10 min, the supernatant was carefully removed, and the tube was inverted to drain briefly on absorbent paper to remove unbound water. At the end of the analysis, the tube containing the wet sediment was weighed again, and the water absorption for each sample was calculated according to Equation (3):

Water absorption (%) = ((W_h_-W_s_)/W_s_) × 100 (3)

where W_h_—wet sediment weight (after centrifugation and drainage); W_s_—initial weight of the dry flour sample (adjusted to the standard moisture basis).

Water activity (*a_w_*) was measured directly at 25 °C using a Novasina LabMaster-aw analyzer (AG, Neuheimstrasse, Lachen, Switzerland), as already described in [31].

Thermogravimetric analyses were performed in a thermogravimetric analyzer TGA55 (TA Instruments, New Castle, DE, USA) in the range of 25 to 800 °C, with a scan of 10 °C·min^−1^, in N_2_ atmosphere (50 mL·min^−1^).

### 2.5. Composition Analysis of Microcapsules

Ash, moisture, protein, lipid and total dietary fiber contents were determined according to the Association of Official Analytical Chemists (AOAC) [32]. Total carbohydrates were estimated by deducting the sum of moisture, ash, protein, lipids and total dietary fiber contents from 100%. Calorific values (kcal) were calculated from the approximate chemical composition data.

Fructose, glucose, sucrose and maltose contents in the beetroot dairy beverage microparticles were evaluated as described in [33], employing an HPLC LC-20AD system (Shimadzu^®^, Chiyoda, Tokyo, Japan) with automatic injector equipped with a NH2 column (5 mm, 250 × 4.6 mm; I.D (Zorbax^®^, Santa Clara, CA, USA). The refractive index detector RID-10A (Waters, MA, USA) coupled to a signal integrator CBM-20A (Shimadzu^®^, Tokyo, Japan) was used. Isocratic elution (82% acetonitrile in distilled and deionized H_2_O) at a 1.0 mL∙min^−1^ flow rate was used.

### 2.6. Extraction and Quantification of Betalains from Microparticles

Betalain extraction and quantification were determined with a modification of the method described in [34]: The extraction was performed in 30% aqueous ethanol previously acidified at pH 5.5 with 1% formic acid. The mixture was homogenized and centrifuged at 15.000× *g* for 30 min, supernatants were collected, and extraction was repeated twice. Then, betalains present in the supernatants were quantified in a UV/Vis spectrophotometer Shimadzu UV 2800 (Shimadzu Scientific Instruments, Inc., Columbia, MD, USA) at wavelengths of 538 nm and 480 nm, to detect betacyanins and betaxanthins, respectively. Betalain content (BT), expressed as betacyanins (BCs) and betaxanthins (BXs), was determined according to Equation (4), as described in [35], using molar extinction coefficient ε = 60,000 L·mol^−1^·cm^−1^ and molecular weight (MW) = 550 g·mol^−1^ for betacyanins, and ε = 48,000 L·mol^−1^·cm^−1^ and Mw = 339 g·mol^−1^, for betaxanthins.

BT (mg·g^−1^) = (A × DF × MW × V)/(ε × L × D × Wd) (4)

where *A*—absorbance at 538 nm and 480 nm for betacyanins and betaxanthins, respectively; *DF*—dilution factor; *L*—cuvette length; *V*—total volume; and *Wd*—microparticle mass.

### 2.7. Encapsulation Efficiency

Encapsulation efficiency (EE) of betalains in samples microencapsulated with cassava starch and maltodextrin was determined individually for each pigment (total betalains, betacyanins and betaxanthins), as described in [36], according to Equation (5):

EE(%) = ((BT − BTS)/BT) × 100.(5)

where BT refers to total betalain content and BTS to betalain content present in the supernatant. EE (%) was determined after dilution of 1 mL sample in 10 mL water and centrifugation, followed by spectrophotometric quantification of betalains (betacyanins + betaxanthins, mg·g^−1^) at 538 nm and 480 nm wavelengths, according to Equation (2). Encapsulation efficiency was determined by the ratio between betalain in the supernatant and betalain content added in the microparticle formulation.

### 2.8. Quantification of Total Saponin, NO_3_^-^ and NO_2_^-^ Contents

Total saponin was quantified by the vanillin–sulfuric acid assay, where the absorbance at 535 nm was recorded using a Jasco V–530 UV/VIS spectrophotometer (Jasco do Brasil, São Paulo, SP, BRA), as described in [33]. The NO_3_^-^ and NO_2_^-^ contents in beetroot dairy beverage microparticles were determined as described in [3]. The HPLC LC-20AD system (Shimadzu Scientific Instruments, Inc., Columbia, MD, USA) with an automatic injector was equipped with a 5 µm reversed-phase C8 column (150× 4.6 mm; I.D, Ascentis, Sigma-Aldrich Chemical Co., Bellefonte, PA, USA) and a 5 µm reversed-phase C18 guard column (50 × 4.6 mm; I.D, Ascentis). The fluorescence detector model RF-10AXL (Shimadzu^®^) was set up at 375 nm excitation and 415 nm emission. Sodium phosphate buffer (pH 7.5) at 15 mmol·L^−1^ and methanol (50:50, *v*/*v*) were used as the mobile phase in gradient elution at a flow rate of 1.3 mL·min^−1^.

### 2.9. Microbiological Monitoring of Beetroot Dairy Beverage over 30 Days

To ensure commercial validity, beetroot formulations were stored in hermetically sealed containers, without vacuum, and analyzed 3 times for 30 days. Counts of molds, yeasts and fungi were performed according to the American Public Health Association (APHA) methodology [37]. The presence of *Salmonella* spp. and *Escherichia coli* was evaluated by the 3M Petrifilm™ Salmonella Express and 3M Petrifilm™ *E. coli* (3M Health Care, Saint Paul, MN, USA) systems, respectively. Total and fecal coliforms were determined by the most likely number per gram (NMP·g^−1^) method, following standard protocols. *Bacillus cereus* was quantified in MYP selective medium (Plast Labor^®^ Rio de Janeiro, RJ, Brazil), according to the APHA.

### 2.10. Sensory Analysis

This study with untrained judges was conducted in accordance with the Declaration of Helsinki and was approved by the Institutional Ethics Committee of Pedro Ernesto University Hospital, Rio de Janeiro (No. 25201019.0.0000.5259, 14 November 2019). About one hundred untrained judges of both genders aged between 18 and 60 years were recruited through invitations posted at the Federal University of Rio de Janeiro (UFRJ) and State University of Rio de Janeiro (UERJ). All participants signed the Informed Consent Form approved by the Institutional Ethics Committee of Pedro Ernesto University Hospital. Participants received instructions for carrying out the tasting and questionnaire answering, accompanied by a researcher who remained throughout the entire procedure to clarify any doubt. Powdered samples were weighed into plastic cups, to which a predetermined amount of chilled filtered water was added. The mixture was homogenized manually with the aid of a spoon until the product was completely dissolved. Each participant evaluated four formulations of microencapsulated beetroot dairy beverage, refrigerated and differentiated by acronyms just after manufacturing. Sensorial evaluations were carried out at 25 °C in temperature-controlled, closed sensory booths with white lighting, and samples were randomly served, accompanied by a questionnaire form. Beetroot dairy beverage formulations were evaluated individually for acceptability by assessing powder attributes, such as color, aroma, taste and texture, and overall impression through a 9-point structured hedonic scale. To encourage non-trained judges to perform a more conscientious purchase intention analysis, purchase intention was scored using a 5-point hedonic scale, ranging from “I would certainly buy it” (score 5) or “I would certainly not buy it” (score 1) [38].

### 2.11. Statistical Analysis

A one-way analysis of variance (ANOVA), with repeated measures, was performed to verify differences resultant from each formulation used in the characterization and stability analyses. When a significant *F* value was identified, a post hoc test with Bonferroni correction was applied. The results are expressed as means ± standard deviations (SDs). All statistical analyses were performed using Graphpad Prism software v. 9 for Windows^®^ (GraphPad Software, CA, USA).

## 3. Results

To evaluate the nutritional and physicochemical properties of the microencapsulated formulations, several physicochemical, biochemical and morphological analyses were performed to monitor the changes promoted by adding encapsulating agents, which are expected to modulate the energy value and the proportion of macronutrients of the product. Data may provide fundamental clarification on the impact of microencapsulation on the food matrix. Furthermore, the calculation and results were expressed considering the finished product, exactly as it is or comes out from the freeze-dryer, including water or residual moisture, reflecting the physical reality of the product; therefore we opted for the more specific method, which is fresh weight bases.

### 3.1. Centesimal Composition and Contents of Sugars and Bioactive Compounds 

The proximate composition and contents of bioactive compounds from the microencapsulated formulations obtained through freeze-drying with or without encapsulating agents are depicted in Table 1. The formulations were microencapsulated in cassava starch, maltodextrin or a combination of 50% maltodextrin and 50% cassava starch (1:1 ratio), and the percentages of moisture and ashes were similar but were lower in the control—powdered dairy beetroot drink (2:1 ratio). As expected, all microencapsulated formulations showed significantly higher carbohydrate content and energy values (*p* < 0.05) than the control—powdered dairy beetroot drink (2:1 ratio)—due to the polysaccharides added, while lipid content was significantly lower in all microencapsulated formulations when compared to the control—powdered dairy beetroot drink (2:1 ratio). Furthermore, protein, lipid and carbohydrate contents in the microencapsulated formulations were similar when compared to each other. Protein, lipid, total dietary fiber, total sugar, fructose, sucrose and glucose contents in the control—powdered dairy beetroot drink (2:1 ratio)—were higher than in the microencapsulated formulations. Total sugars (23.96 ± 1.66 g·100 g^−1^), maltose (4.75 ± 0.11 g·100 g^−1^) and glucose (3.26 ± 0.10 g·100 g^−1^) in the formulations microencapsulated in maltodextrin were higher than those in the formulations microencapsulated in cassava starch and in 50% maltodextrin combined with 50% cassava starch (1:1 ratio). Critically, the concentration of the target bioactives, i.e., saponins (>2000 mg·100 g^−1^) and NO_3_^-^ (>10 mmol·100 g^−1^/620 mg·100 g^−1^), in the microencapsulated formulations were higher, but smaller than in non-microencapsulated dairy–beetroot powder, but the results did not differ among the microencapsulated formulations.

### 3.2. Morphology of Dairy–Beetroot Powder Drink Microparticles

**Figure 1:**
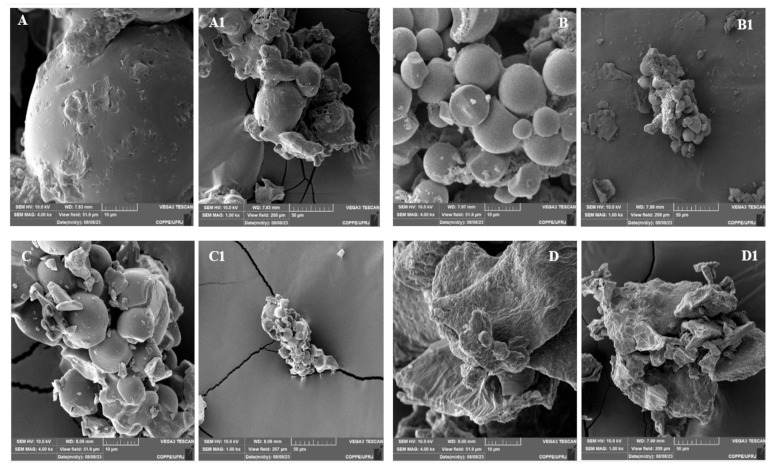
Representative micrographs of microparticles from the microencapsulated formulations prepared through freeze-drying with or without polysaccharide encapsulating agents at 4000× and 1000× magnifications, respectively. (**A**,**A1**) Dairy–beetroot powder drink microcapsules prepared with maltodextrin in 2:1:1 (*w*/*w*) ratio; (**B**,**B1**) dairy–beetroot powder drink microcapsules prepared with cassava starch in 2:1:1 (*w*/*w*) ratio; (**C**,**C1**) dairy–beetroot powder drink microcapsules prepared with 50% cassava starch and 50% maltodextrin in 2:1:1 (*w*/*w*) ratio; (**D**,**D1**) dairy–beetroot powder drink without polysaccharides.

Representative micrographs of the microencapsulated formulation with or without polysaccharide encapsulating agents are depicted in Figure 1, with emphasis on the structural differences observed among the formulations. The SEM images of microencapsulated formulations show that maltodextrin presented a slightly glassy appearance but with a spherical tendency, without invagination or roughness (Figure 1A). The formulations microencapsulated in cassava starch presented spherical and irregular geometric conformations but without cracks or roughness (Figure 1B). A better morphology was observed for dairy–beetroot powder drinks microencapsulated in 50% maltodextrin and 50% cassava starch (1:1 ratio), since microparticles showed the spherical shape characteristic of cassava starch and the glassy appearance of maltodextrin (Figure 1C). The SEM images of the control—powdered dairy beetroot drink (2:1 ratio)—with no encapsulating matrix, even with the addition of large amounts of dairy bases, show no visible microparticles, leading us to conclude that without polysaccharides, this formulation shows a very irregular structure with no defined morphology. The SEM images of the control— powdered dairy beetroot drink—differ from the others, showing regular and spherical structures (Figure 1D).

### 3.3. Microparticle Distribution and Size 

Microparticle size distribution was determined to evaluate the performance and stability of the formulations. The characteristic diameters in the ranges of d (0.1), d (0.5) and d (0.9) revealed significant differences between the formulations with or without polysaccharides, revealing the effect of the encapsulating materials on the microencapsulating structures (Table 2). Remarkably, dairy–beetroot powder drink microencapsulated in maltodextrin showed a reduction in the d (0.5) diameter range, reaching values varying from 15 to 16 μm, which represents a 10× decrease when compared with the control. This difference was validated statistically, since control and formulation containing only cassava starch exhibited larger sizes in the three ranges considered. In general, particle homogeneity, determined by the *span* index, was also variable, even though the dairy–beetroot powder drink microencapsulated with maltodextrin and cassava starch showed the highest *span* (18.94 ± 0.56), because in most formulations, a predominant population of small microparticles predominated, which may have favored rehydration and solubility parameters.

### 3.4. Fourier Transform Infrared Spectroscopy (FTIR) of Microparticles

**Figure 2:**
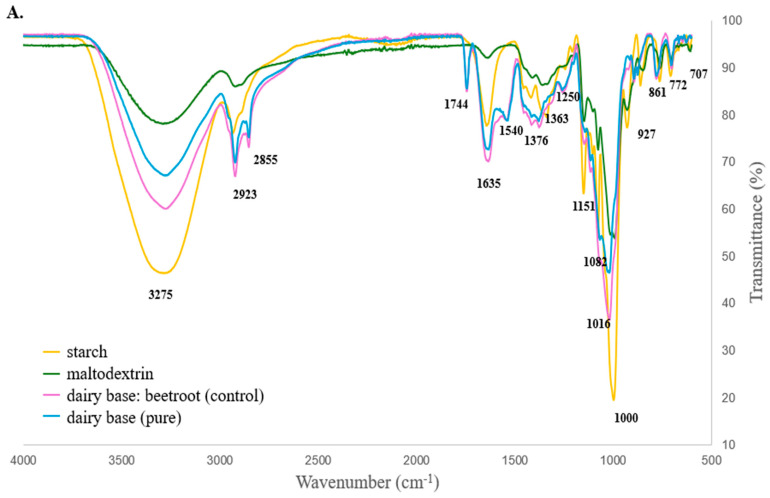

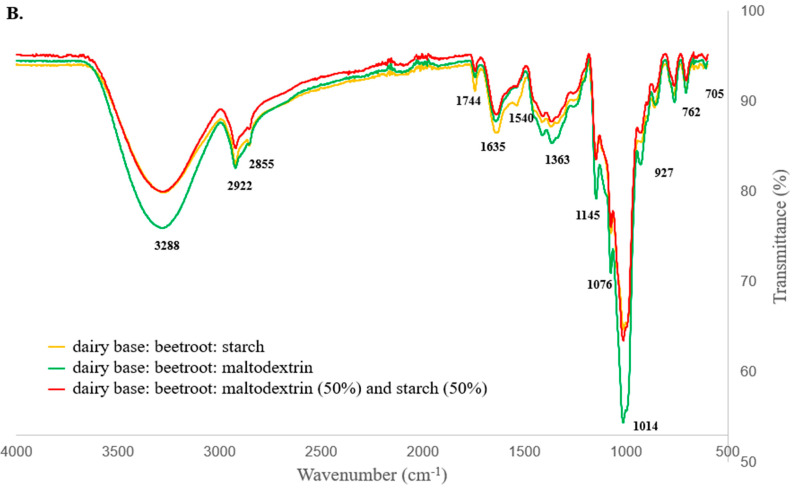
Panel (**A**): FTIR of individual encapsulating agent: cassava starch (in yellow), maltodextrin (in green), beetroot dairy beverage without any encapsulating agent (in purple) and pure dairy base (in blue). Panel (**B**): FTIR spectra of the microencapsulated formulations in: cassava starch (in yellow), maltodextrin (in green), and mixture of 50% cassava starch and 50% maltodextrin in 1:1 ratio (in red).

The FTIR analyses enabled the identification of the major functional groups of the materials and were used to evaluate the interactions between the encapsulating agents and the compounds in the microencapsulated formulations within the microcapsules. The infrared spectra of cassava starch, maltodextrin, dairy base beetroot and dairy base are shown in Figure 2A. The FTIR spectrum of cassava starch shows a broad band in the range of 3500–3000 cm^−1^, corresponding to the stretching vibration of O–H bonds. The band at 2923 cm^−1^ corresponds to the C–H stretching vibration of the polymeric chain [39]. At 1635 cm^−1^, the water band present in cassava starch is observed, indicative of the substance’s high hydrophilicity [40]. Bands between 1700 and 900 cm^−1^ are referred to glucose units, i.e., the monomeric building blocks of amylose and amylopectin, which are the main components of cassava starch [41,42]. The bands at 1082 and 1000 cm^−1^ are attributed to C–O stretching vibrations, while the bands at 929, 861, and 772 cm^−1^ correspond to the C–O–C stretching vibrations of the carbohydrate ring [40].

As expected, the maltodextrin FTIR spectrum (Figure 2A) shows characteristic bands from its oligomers. The broad band at 3500–3000 cm^−1^ corresponds to O–H stretching vibration, and the band at 2923 cm^−1^ corresponds to the C–H stretching vibration of the alkyl group [43]. The characteristic bands at 1635 cm^−1^ and 1358 cm^−1^ can be attributed to the angular deformation of the OH bond (due to absorbed water) and the angular formation of the C-H bond, respectively. In the region of characteristic carbohydrate absorption bands, it is possible to observe bands at 1151 cm^−1^, 1082 cm^−1^ and 1000 cm^−1^ referring to the deformations of C-O and C-O-C bonds [44]. In the fingerprint region, the bands at 707, 772 and 927 cm^−1^ refer to pyrane ring vibrations [45]. It is worth noting that maltodextrin is produced through the partial hydrolysis of cassava starch, with both being glycosidic carbohydrates, and this similarity in chemical composition is evidenced by the similarity in the infrared spectra of maltodextrin and cassava starch (Figure 2A). After encapsulating the beetroot dairy beverage in three distinct polymeric matrices—cassava starch, maltodextrin and an equal-part combination of cassava starch and maltodextrin (1:1 ratio)—a new FTIR spectrum was obtained (Figure 2B). All microcapsules predominantly exhibit the bands mentioned above for the individual polymers used as encapsulants, i.e., cassava starch and maltodextrin.

### 3.5. Physicochemical Properties

The yield, *a_w_*, pH, solubility and absorption indexes of the microencapsulated formulations are shown in Table 3. No significant differences were observed for the microencapsulated formulations concerning encapsulating agents, ranging from 64.74% to 68.02%, showing satisfactory yields. Critically, the formulation with no encapsulating polysaccharide obtained significantly reduced yield (21.41%), demonstrating the importance of wall polysaccharides in the retention of solids during and after the freeze-drying process. Remarkably, powdered dairy beetroot drink microencapsulated in cassava starch and in the combination of 50% maltodextrin and 50% cassava starch presented very low *a_w_*, 0.21 ± 0.03 and 0.22 ± 0.02, respectively. The highest water solubility index was found in microencapsulated formulations, ranging from 91.02 ± 2.71 to 97.86 ± 2.30%. No differences were observed in dairy–beetroot powder drink microencapsulated in cassava starch or maltodextrin; both were higher than 87%. When no encapsulating agent was added, the control—powdered dairy beetroot drink (2:1 ratio)—presented a lower water solubility index. However, the powdered dairy beetroot drink (2:1 ratio) presented the highest water absorption indices (212.04 ± 2.37%), and in those formulations that incorporated maltodextrin as the encapsulating agent, lower water absorption indices were observed (72.42 ± 1.82% and 113.37 ± 1.48%). Furthermore, pH did not vary among all microencapsulated formulations, remaining slightly acidic, ranging from 4.66 to 4.85.

### 3.6. Thermogravimetry (TGA)

**Figure 3:**
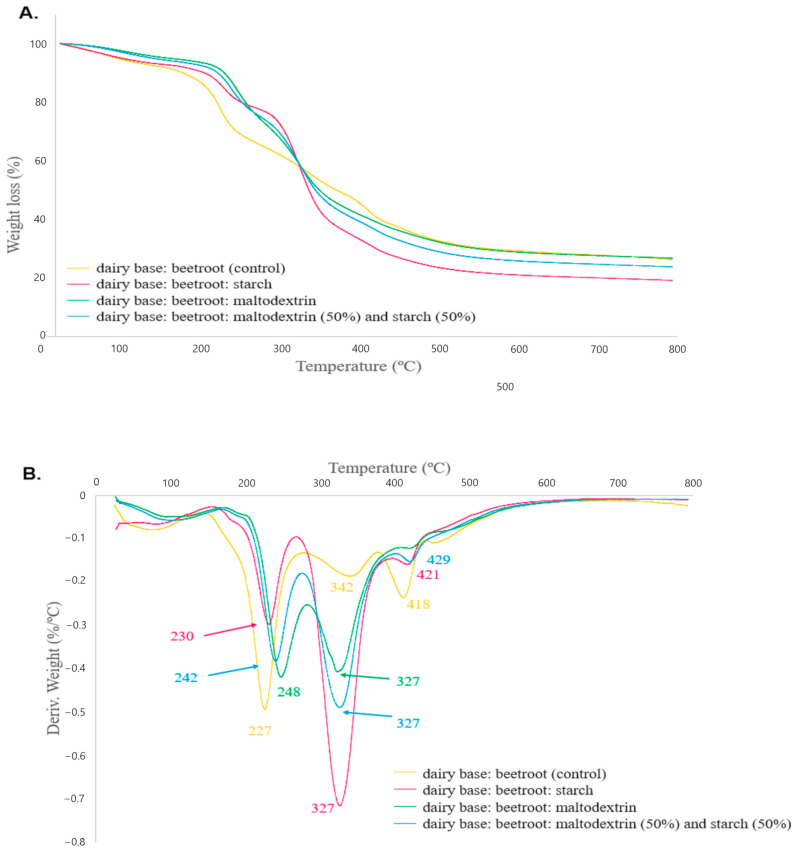
(**A**) TGA curves and (**B**) DTG curves of non-microencapsulated formulations (in yellow), powdered dairy beetroot drink microencapsulated in starch (in pink), in maltodextrin (in green) and in starch–maltodextrin mixture (in blue).

Loss weight and thermal stability of the microencapsulated formulations are depicted in Figure 3. The thermograms show the mass loss (%) variation of the samples according to the temperature (Figure 3A), and the derived thermogravimetry curves, which correspond to the derivative of this curve, highlight the rates of mass loss or gain, facilitate the identification of specific thermal events and allow for drawing inferences about the thermal stability of the microparticles (Figure 3B). All microencapsulated formulations exhibited similar mass loss behavior as a function of temperature, with two or three stages of decomposition occurring at different temperatures. The microparticles presented the first mass loss event (approximately 7%) at 50–150 °C, referring to the loss of water adsorbed on the material. In fact, the presence of the OH bond was confirmed by FTIR analysis by a broad band in the region of 3500–3000 cm^−1^ (see Figure 2), which can be attributed to the surface moisture present in the microcapsules. The second stage, at 170–270 °C, refers to the beginning of the disintegration and decomposition of proteins and carbohydrates from milk and beetroot, and this event showed 27% mass loss for powdered non-microencapsulated formulations, which was superior when compared with formulations microencapsulated in starch, maltodextrin, and maltodextrin combined with cassava starch, presenting mass losses of 15%, 21% and 18%, respectively. The experiment was completed at 800 °C with a residual mass of 30% for the powdered non-microencapsulated formulations and 27%, 41% and 33% for powdered dairy beetroot drink microencapsulated in starch, in maltodextrin, and maltodextrin combined with cassava starch, respectively.

### 3.7. Efficiency of Encapsulation of Betacyanins, Betaxanthins and Total Betalains

The EE of microencapsulated formulations is shown in Table 4. According to the final concentration of compounds per gram of microcapsule, the best results were obtained for cassava starch and mixed polymers as the encapsulating agents, as they retained the highest contents of betacyanins, betaxanthins and total betalains when compared with microencapsulation using maltodextrin or powdered non-microencapsulated formulation. Furthermore, all microencapsulated formulations showed EE higher than 63% for total betalains, confirming the viability of the microencapsulation method. However, we observed clear differences among the polysaccharide coatings. Remarkable, the combination of maltodextrin and cassava starch as the encapsulating agent showed the highest overall EE for total betalains (81.75%), betacyanins (80.12%) and betaxanthins (78.76%), when compared with the other treatments. Cassava starch, used as the encapsulating agent, showed intermediate EE performance of 71.28%, 72.53% and 74.41% for betacyanins, betaxanthins and total betalains, respectively, whereas maltodextrin as the encapsulating agent showed the lowest EE, lower than 65% for all compounds.

### 3.8. Microbiological Stability of Microparticles

During the manufacturing and storage of the microencapsulated formulations, all procedures and analyses were performed according to IN 60/2019, in addition to the RDC 331/2019 norm by the current Brazilian legislation (ANVISA, 2019). Microbiological analyses revealed that the microencapsulated formulations meet the microbiological parameters established by national and international regulations (ANVISA, 2019), indicating that they are safe for human consumption for at least 30 days even when storing the microparticles at room temperature (Table 5). Furthermore, microbiological analysis findings of dairy–beetroot powder drink microencapsulated in maltodextrin, in cassava starch and in a combination of 50% maltodextrin and 50% cassava starch (1:1 ratio) show that all microbiological parameters results are lower than for the non-microencapsulated formulation. However, after this period, molds, yeasts and fungi were detected in the formulations, suggesting that the microorganisms from the fermentation stage remained viable, preserved by microencapsulation processing, yet were able to exert positive effects on gastrointestinal colonization. It should be noted that there are no specific safety guidelines for molds, yeasts and fungi in powdered foods, since the presence of pathogenic species has not been reported in this type of food matrix; therefore, it causes no concerns in terms of contamination or safety failure. However, in the case of dairy products, the technological complexity and the addition of ingredients susceptible to spoilage, such as milk and whey, may reduce shelf life even under refrigeration. Thus, the shelf life of the microencapsulated formulations indicates adequate hygienic–sanitary processing conditions, but the shelf life could potentially be increased through vacuum packaging.

### 3.9. Sensory Analysis Results

**Figure 4:**
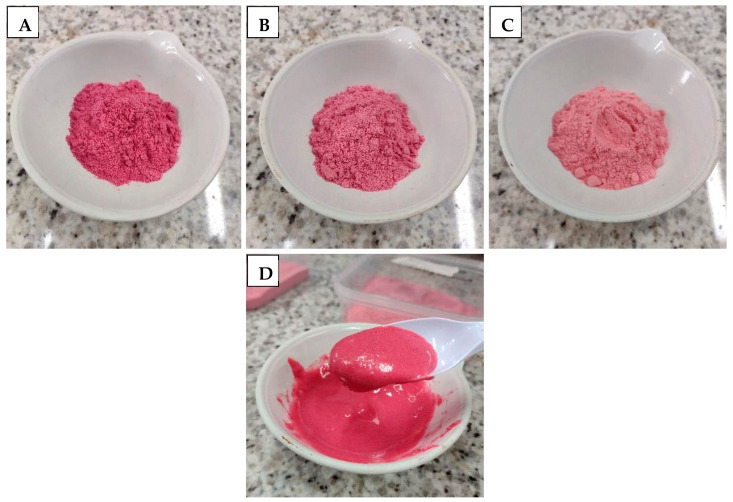
Beetroot dairy drinks microencapsulated in polysaccharide encapsulant agents and the final product reconstituted in ice water, ready for consumption. (**A**) Dairy base: beetroot: maltodextrin; (**B**) dairy base: beetroot: maltodextrin (50%) and starch (50%); (**C**) dairy base: beetroot: starch; (**D**) beetroot drink rehydrated in ice water ready for consumption.

The microencapsulated formulations (Figure 4) were evaluated for their sensory attributes and overall impression in an acceptance test, following the requirement for the development of innovative foods (Table 6). Samples were first reconstituted in ice water following a standard like those for commercial powder products: an amount of 50 g of the product was dissolved in 150 mL of water, resulting in a smooth and homogeneous final product of paste consistency, without any lumps (Figure 4D). Notably, dairy–beetroot powder drink microencapsulated in maltodextrin and in the 50% maltodextrin and 50% cassava starch combination exhibited the best texture, taste, overall acceptability attributes and purchase intention when compared with dairy–beetroot powder drink microencapsulated in cassava starch and formulation with no encapsulating polysaccharide agent. Color was the attribute which received average scores corresponding to “I liked it moderately” (score ≥ 7.0), indicating high product acceptance for all microencapsulated formulations with or without encapsulating agent. Furthermore, only dairy–beetroot powder drink microencapsulated in maltodextrin and in the 50% maltodextrin and 50% cassava starch combination received average scores corresponding to “I liked it moderately” (score ≥ 7.0) in texture and taste attributes and overall acceptability. In addition, dairy–beetroot powder drink microencapsulated in maltodextrin and in the combination of maltodextrin and cassava starch received the highest purchase intention scores, corresponding to “I would probably buy it” (score ≥ 4.0).

## 4. Discussion

Dairy–beetroot powder drink microencapsulated in cassava starch, maltodextrin or in a combination of them showed extremely low moisture contents, under the limit (<15%) established by the current Brazilian sanitary legislation for powdered foods [46]. This is a desirable condition since high moisture in powdered foods can favor spoilage processes, with a negative impact on sensory attributes and overall product acceptability [47].

UHT whole milk, one of the dairy base components, was the main contributor to the lipid content found in products with and without encapsulating agent, consisting of triglycerides, phospholipids, and cholesterol [48]. However, all microencapsulated formulations showed reduced lipid content (<11%), because the microencapsulated products were formulated with a minimum dairy base of 51% in accordance with the guidelines of Normative Instruction No. 16/2005 of MAPA [25]. Heterogeneous retention or distribution can happen by physical trapping: when the matrix (e.g., maltodextrin, gum Arabic, or protein isolates) forms a dense network, the solvent cannot dissolve this network, and the internal lipid remains untouched. Furthermore, regarding incomplete encapsulation vs. efficiency, the traditional AOAC method often quantifies only the surface lipid (free oil), ignoring the effectively encapsulated oil if there is no prior acid or base hydrolysis step [49].

Although widely used as encapsulating agents in the food industry due to their solubility, stability, and low price, maltodextrin and cassava starch are not considered nutritionally neutral and contribute to an increase in carbohydrates and energy in microencapsulated formulations. Maltodextrin is a glucose polymer obtained from the controlled hydrolysis of starch, and starch is organized in granular form, composed of amylose and amylopectin macromolecules, with both being high-molecular-weight carbohydrates [50]. Herein, microencapsulation using polysaccharides increased carbohydrate content and total energy of all microencapsulated formulations, and the final characteristics of the product should be considered in the nutritional declaration, especially for those products intended for carbohydrate-restricted diets.

The dairy–beetroot powder drink microencapsulated in cassava starch, in maltodextrin or in combination of them showed high content of fiber, over 3 g∙100 g^−1^ of product, being considered a fiber food source, in accordance with current Brazilian legislation [51]. High intake of dietary fiber is widely recognized as conducive to reducing risks of coronary and cardiovascular diseases, as well as contributing to lower blood cholesterol, either by increasing the secretion of bile acids or by producing short-chain fatty acids; inhibiting the growth of pathogenic bacteria; and stimulating the development of microbiota, which strengthens the immune system responses and helps prevent gastrointestinal infections [52].

The high protein content (>19%) of the microencapsulated formulations is in accordance with Brazilian laws, exceeding this expectation, standing at 13% protein (Normative Instruction MAPA 28/2007) [53]. The protein amount in the microencapsulated formulations reached the expected level, according to the formulation itself, since it was prioritized to keep a dairy base of 51%, according to the guideline of Normative Instruction No. 16/2005 of MAPA [25]. The microencapsulated formulations both present the protein concentration intake characteristics of a food protein source and contain several other compounds with functional attributes capable of acting as adjuvants in human diets.

About 85% of NO_3_^-^ intake in Western diet comes from vegetables, and beetroots contain higher NO_3_^-^ content and are commonly used for dietary interventions to improve cardiovascular function by decreasing blood pressure and arterial stiffness, improving overall vascular function [4,5,11,54]. Dietary NO_3_^-^ supplementation should last over 20 days and be greater than 370 mg (or 6.0 mmol·day^−1^) to promote NO and improving the hemodynamic and vascular benefits in those individuals with cardiovascular risk factors [54,55,56,57,58]. However, as already mentioned, the high beetroot amounts needed to achieve effective dietary NO_3_^-^ concentrations in the human body limit adherence to long-term nutritional interventions [54]. In the present study, a novel microencapsulated formulation technology overcomes the challenge of developing a high-acceptance beetroot formulation containing pharmacological concentrations of NO_3_^-^ content (>615 mg, corresponding to 10 mmol·100 g^−1^) in all microencapsulated formulations in an adequate serving portion.

Saponins are complex compounds that vary extensively in structure and are widely distributed in edible vegetables, exhibiting antiallergic, antiviral, antimicrobial, anti-inflammatory, antioxidant, antidiabetic and antihemolytic properties, depending on their type and concentration. Saponins from *Beta vulgaris* L. are triterpene glycosides, where the aglycone—hederagenin, akebonoic acid, oleanolic acid or gypsogenin—is covalently linked to monodesmoside or didesmoside sugar chains through a glycoside ester-to-C-28 or ether-to-C-3 bond [59]. Saponins are found in the leaves and roots of red beetroots, as well as in sugar beetroots, forming a great natural diversity of saponins [59,60]. The complete structural characterization of saponins in plant materials is complicated and time-consuming. A few studies in red beetroots have identified and quantified fourteen triterpene saponins derived from oleanoic acids by employing reverse-phase liquid chromatography coupled with electrospray ionization mass spectrometry (LC-ESI/MS/MS). Saponin content may change according to the cultivar, with concentrations varying from 766 to 1220 mg·100 g^−1^ dry weigh in three different cultivars of *Beta vulgaris* L. [59]. The authors of [61] identified twenty-four triterpene saponins from oleanolic acid, hederagenin, akebonoic acid and gypsogenin as aglycones in extracts of fresh peel and flesh from four red *Beta vulgaris* L. cultivars, and the saponins ranged from 106 to 683 mg·100 g^−1^ fresh weigh in the peel and from 92 to 686 mg·100 g^−1^ fresh weight in the flesh of beetroots. Saponin content may also vary according to beetroot processing: in the study conducted in [7], saponin content was almost three times higher in beetroot gel when compared with juice, 2200 and 822 mg·100 g^−1^ fresh weight, respectively. Herein, the high saponin content (>2000 mg·100 g^−1^ fresh weight) in all microencapsulated formulations is due to freeze-drying technology, which promoted the concentration of all nutrients and bioactive compounds from beetroot following water removal by sublimation, with no increase in temperatures that could degrade sensitive molecules. The process employed herein reduces the sample mass, preserving the chemical integrity of phytochemicals, including saponins, and increasing compound concentrations on a dry weight basis. The technique proved to be an efficient strategy for enhancing the nutritional and functional density of microencapsulated formulations [62].

Microparticle morphology provided important information about the influence of polysaccharides on the final characteristics of formulations. No cracks or open pores were observed in all microparticles. However, their morphology varies according to the polysaccharide used to build them, which affects their efficacy and stability for microencapsulation of active food compounds. When cassava starch was used, the microcapsules presented spherical and irregular geometric conformations, in contrast to the maltodextrin microparticles, which presented a slightly glassy appearance but displayed a spherical tendency. The geometric structure, surface porosity, and integrity of the microcapsules are crucial characteristics, as they influence the core protectability, release rate, and interactions between the matrix and the core. Furthermore, microparticle microstructure, size, polydispersity and morphology may undergo changes during freeze-drying depending on the encapsulating matrix composition and properties, the core: wall ratio, and the drying and storage conditions [63].

The size of microparticles exerts a direct influence on their functional properties, which, together with other characteristics, such as dispersibility, solubility, and water activity, influence the stability and the kinetics of release of bioactive compounds within the core [64]. Furthermore, larger microparticles offer greater protection to the core material but have lower dispersibility, while very small microparticles are more soluble, but the core material may be absent or in very low concentrations, impairing process efficiency [65]. The present study employed the laser diffraction method to analyze the average particle size of microparticles, measuring time-dependent fluctuations in scattered light, i.e., the decay of the autocorrelation function caused by the diffuse motion of the particles and the experimentally measured diffusion coefficients that were mathematically converted into hydrodynamic diameters [66]. Microparticle size distribution and the scattering index (“*span*”), which expresses the amplitude of variation between the diameters, observed in all formulations with polysaccharides as encapsulating agents in the present study exhibited sizes lower than 100 μm in the range of d (0.5) for each sample, indicating that the diameter of polysaccharide particles is in the micrometric scale and in a similar range of other microparticles already built, such as the roasted coffee oil microencapsulated in maltodextrin microcapsules through freeze-drying [67]. It should be noted that microparticle size could be influenced by the coating compounds used in the encapsulation process, although they also impact the sensory properties of the final product and the solubility of the mixture during the reconstitution of the final product summed to the moisture, flow behavior, compaction, and rehydration capacity of the microencapsulated material [68]. Among the encapsulating agents tested, formulations built with maltodextrin showed smaller particle sizes compared with the others, displaying a narrower distribution even with greater *span*. Keeping a regular size in microparticles improves the reconstitution of the freeze-dried product.

The *span* index was used to infer the width of the particle size distribution and served as a direct indicator of homogeneity, since a low *span* index denotes a narrower distribution and more homogeneous particles, while a high *span* index denotes a wider distribution and particles of varying sizes coexisting in the same powder. A good *span* index value will depend on the technological objective. For applications seeking uniformity and stability, lower *span* values are desirable. In food systems, such as powdered dairy beverages, a higher *span* index is not necessarily bad and may indicate the coexistence of small and large particles, which, together, favor functional properties [66,68]. In the present study, the microencapsulated formulations with maltodextrin and cassava starch showed the highest *span* index compared with formulations with only one encapsulant agent, which may have created a more porous structure or differentiated dissolution channels, favoring hydration and solubility [65,68]. Furthermore, the d (0.5) values of the microencapsulated formulations with maltodextrin and cassava starch were low, indicating a predominance of fine microparticles. Therefore, even though it is less homogeneous (high *span*) and the population is mostly composed of small particles, it compensates for the heterogeneity, and the system also benefited from larger and smaller particles acting together to accelerate dispersion in water [65,66,68].

The FTIR control consisted of a mixture of beetroot and dairy base. A broadband was identified in the 3500–3000 cm^−1^ region, referring to the stretch of the O-H bond. The bands at 2923 and 2855 cm^−1^ show the stretching of the C-H bond of the aliphatic chain [69]. At 1744 cm^−1^, a band referring to the stretching of the carbonyl bond, C = O, was detected, which can be associated with both the fatty acids present in the milk drink [69,70] and the carboxylic acid of betalains, the main pigment of beetroots [71,72]. At 1635 cm^−1^, a band characteristic of the stretching of the imine bond (C = N) of betalains was observed [27,73,74], but it can also be attributed to the stretching of the C = O amide bond present in the peptide backbone of milk proteins [69]. The band at 1540 cm^−1^ has also been reported [75,76] as referring to the flexion of the N-H bond associated with the amide function, also found in the structure of milk proteins. The broad band at 1080–1010 cm^−1^ refers to the stretching of the C-O bond present in the carbohydrate structures that make up beets and the milk base [76,77]. However, small variations are noted in the intensity, shape and values of the FTIR bands of the isolated materials, when compared with building microcapsules, which may be the result of intermolecular interactions between the beet and dairy beverage and the coating polymers after microencapsulation [78]. In addition, the presence of the carbonyl band at 1744 cm^−1^ exclusively in both beetroot and dairy components and its absence in the spectra of the pure encapsulating agents serve as strong pieces of evidence that the core beetroot–dairy materials were successfully incorporated and preserved in the microcapsules [79,80].

Microencapsulation yield increased with the increase in encapsulating agent concentration and solid contents, indicating that a successful drying process occurred. The addition of each encapsulating agent may achieve similar yields, because a unique proportion (ratio 2:1:1) was applied to all microencapsulated formulations, even though the molecular structure of cassava starch may favor higher yield compared with maltodextrin.

Regarding *a_w_*, all formulations tested should be considered safe because their *a_w_* ≤ 0.3 does not favor the growth of pathogenic and deteriorating microorganisms that could synthetize hydrolytic enzymes to break down substrates for microbial growth [81]. The *a_w_* values found herein are like those of freeze-dried buriti oil microparticles for yogurt enrichment, which ranged from 0.27 to 0.38 [82], or those of the microcapsules of mulberry extract obtained through freeze-drying, which reached *a_w_* = 0.5 [83].

*a_w_* should be probably the most critical parameter for the stability of food powders, especially freeze-dried powders, which are highly hygroscopic (absorb moisture easily). In the case of dairy–beetroot powder drink, the interaction between the milk proteins and the sugars/pigments of the beetroot makes the powder very sensitive. Products exhibiting *a_w_* ≤ 0.3 fall within the “safety zone” for dehydrated foods. For a freeze-dried dairy–beetroot powder drink, this moisture level represents the ideal scenario, where physical and chemical stability is maximized [84]. Water acts as a plasticizing agent, and when *a_w_* ≤ 0.3, the amount of available water is extremely low and insufficient to act as an effective plasticizer. With so little water, the glass transition temperature of the mixture (milk + beetroot + encapsulant) remains above the room temperature, and the dairy–beetroot powder remains in an amorphous, glassy state, where viscosity is extremely high and molecular mobility is almost zero. This condition prevents particles from “deforming” or creating sticky contact points [85]. Encapsulant agents such as maltodextrin have a naturally high glass transition temperature, which helps to keep the powder stable. Starch (especially modified starch) acts similarly, but its semi-crystalline structure may offer a different physical barrier against moisture [86,87,88].

Maltodextrin is an excellent carrier agent for freeze-drying due to its high solubility and film-forming ability. At *a_w_* ≤ 0.3, the tendency to clump is minimal, and the powder is dry to the touch, does not adhere to stainless steel or glass surfaces, and exhibits high fluidity [87]. Starch (especially if it is modified or pre-gelatinized starch) acts as a more robust bulking agent. It exhibits lower hygroscopicity than maltodextrin, and even with slight fluctuations in relative humidity, starch holds up well without forming lumps. It has good flowability, but this may be slightly lower than that of pure maltodextrin due to the morphology of the particles, which tend to be larger and less regular, potentially increasing interparticle friction [88]. Furthermore, the dairy–beetroot powder microencapsulated with a blend with 50% starch and 50% maltodextrin is, theoretically, the most balanced formulation because maltodextrin offers a chemical barrier and high solubility, while starch provides a structural “backbone” that better supports increased *a_w_*. Furthermore, this blend promotes structural synergy, where maltodextrin fills the spaces between the starch chains, creating a dense and protected matrix. Agglomeration is virtually nonexistent at *a_w_* ≤ 0.3, and the resulting powder is very stable and “loose.” This blend typically exhibits very consistent flow behavior, making it ideal for industrial packaging processes [86,88].

Betalains, the beetroot pigments, are extremely sensitive to oxygen, light, and especially to humidity and temperature. At low *a_w_* likely observed in the present formulations, the chemical degradation of betalains by hydrolysis and oxidation should be minimal. In the glassy matrix formed by the encapsulating agent, the pigment is trapped in a kind of molecular cage, and without free water to facilitate chemical reactions, the degradation rate of this pigment and antioxidant compound is drastically reduced. Maltodextrin creates a very efficient glass barrier that retains betalains and protects them against oxygen exposure more effectively than pure starch. Starch can create a more porous and opaquer matrix, which protects betalains against photodegradation (light), and although it offers less protection against microscopic oxidation, it maintains the physical integrity of powder for longer [87,89,90,91]. In a blend with 50% starch and 50% maltodextrin, theoretically, the chemical protection of maltodextrin against betalains occurs, along with the physical resistance of starch against clumping, which indirectly protects betalain from being exposed by the collapse of the powder. Therefore, the blend stands out not only for its immediate stability but also for its resilience, and if there is a packaging failure and humidity rises slightly, starch will delay the disaster (clumping), while maltodextrin protects the pigment.

The water solubility of freeze-dried products is a relevant technological attribute, often associated with consumer’s acceptance, and should be considered for the selection of the encapsulating agent. In this context, polysaccharides are recommended to encapsulate matrices, since they form microparticles with high redispersion capacity and posterior solubilization in aqueous media [92]. Herein, an increase in formulation solubility was observed after microencapsulation in maltodextrin, starch, or their combination (≥90%), indicating rapid and almost complete dissolution. However, proteins can suffer losses during the dehydration process, thus reducing their solubility [93]. In microencapsulated formulations intended for reconstitution in aqueous media, reduced values of the water absorption index are preferable, since they indicate lower water retention in the matrix, favoring the fast solubility of the product. On the other hand, high values of the water absorption index can lead to the formation of clumps when in contact with water and, consequently, hinder dissolution process. The best result considering this parameter was reached by employing cassava starch (close to 70%), showing high dispersion capacity of the formulation in liquid medium. It is likely that this behavior is related to the commercial cassava starch used and not native cassava starch. Corn starch was already used as an encapsulant in a freeze-dried beet formulation and had a water absorption index of 220% [27]. The results obtained here are also close to the values of 153–173% of the water absorption index obtained for freeze-dried kiwi pulp [94].

All microencapsulated formulations with and without encapsulating agents presented acid pH without differences. The pH plays a crucial role in the solubility of powdered dairy beverages by directly influencing the interaction of proteins with water. When the pH values are close to 4.6, it can reduce solubility due to protein aggregation, while more alkaline pH increases the negative charge, favoring the dissolution of the powder. These effects are relevant in freeze-drying, as rehydration is heavily dependent on the pH of the formulation [95]. The bacteria present in kefir culture added to the microencapsulated formulations use both homofermentative and heterofermentative routes, resulting in the accumulation of organic acids, which translates into titratable acidity [31]. Furthermore, acidity is one of the most effective defense mechanisms of fermented dairy beverages, as it inhibits pathogenic and spoilage microorganisms (such as *Salmonella* or *E. coli*). The acidic environment created during fermentation, resulting from the breakdown of sugars into organic acids, creates a hostile environment for the survival and multiplication of microorganisms. These acids, such as lactic acid and acetic acid, stimulate cell formation and microorganism reproduction, contributing to the preservation and safety of food products. In addition, the product developed in this study is in powder form, and this residual acidity aids in natural preservation, extending shelf life and ensuring that the product is safe for consumption [96].

The acidic pH alters the chemistry of the minerals present in milk (such as calcium, magnesium, and phosphorus), helping to solubilize these minerals and transforming them into forms that the human body can absorb much more easily in the digestive tract compared with unfermented milk. The acidic pH is close to the isoelectric point of some milk proteins, such as casein, aiding in their digestion in the human body. Therefore, some milk proteins have already undergone partial denaturation and fine precipitation during fermentation. For the consumer, this translates into a beverage that is more quickly and easily digested, reducing the bloating sensation often associated with the consumption of conventional dairy products [96,97,98].

Acidic chyme can help to reduce the glycemic response through a combination of chemical and enzymatic mechanisms that occur from the stomach to the small intestine. When the stomach contents are too acid, due to the presence of organic acids (especially lactic and acetic acid), as it occurs after ingesting a fermented beverage, the body slows gastric emptying to allow the pancreas to neutralize the acid chyme efficiently. As a result, food takes longer to pass from the stomach to the small intestine (where glucose absorption occurs), and glucose enters the bloodstream slowly and gradually, preventing insulin spikes [99,100,101]. The acidic pH and the presence of organic acids generated during fermentation can reduce the activity of alpha-amylase and alpha-glucosidase enzymes, reducing the hydrolysis of carbohydrates into glucose and resulting in a lower glycemic load available for immediate absorption [101,102,103]. Furthermore, acidic fermented foods, especially containing lactic acid, can stimulate the L cells from the intestine to secrete GLP-1. This hormone not only improves insulin secretion by the pancreas in a controlled manner but also helps to suppress glucagon and increase the feeling of satiety, which indirectly improves fasting glucose and glycated hemoglobin levels and reduces visceral fat and body mass index through appetite modulation while reducing total cholesterol and LDL-c [104,105,106].

In addition to lower mass loss, the microencapsulated formulations with starch (230 °C), 50% maltodextrin combined with 50% cassava starch (242 °C) and maltodextrin (248°C) exhibited the highest maximum temperatures, showing a subtle increase in the thermal stability of the control drink compared with the microencapsulated products, where lower mass loss was recorded for the starch microcapsule and the highest initial degradation temperature was recorded for the maltodextrin polymer matrix. Furthermore, the third stage occurs between 270 and 380°C and presents a mass loss of 44% for the microencapsulated formulations with starch, 32% for maltodextrin and 36% for maltodextrin combined with cassava starch (1:1 ratio). This event, with a maximum temperature of 327°C for all microencapsulated formulations, is attributed to the degradation and thermal decomposition of starch polysaccharides and maltodextrin oligosaccharides [107,108]. Wall material degradation results in a significant reduction in active ingredients in microcapsules, inferred by the loss of masses [109]. In addition, at temperatures above 380°C, only a small mass loss was observed for all microencapsulated formulations, which can be explained by the decomposition of the remaining organic compounds and ash formation from microcapsules [110].

The TGA technique applied to microencapsulated foods with polymers such as starch and maltodextrin is of great scientific and technological relevance, as it provides detailed information on thermal stability, encapsulation efficiency, and the behavior of materials under processing conditions. Through TGA, it was possible to identify distinct stages of mass loss, such as initial dehydration, degradation of encapsulating polymers, and controlled release of bioactive compounds. These data allow us to understand how starch and maltodextrin act in protecting sensitive nutrients from beetroot, such as antioxidant compounds, ensuring that the polysaccharides confer greater heat resistance during industrial processes or prolonged storage. Furthermore, the technique aids in comparing different encapsulating agents, guiding the selection of more suitable materials for specific applications and contributing to the development of functional foods adding quality, stability, and value [111]. In the present study, as demonstrated by the TGA technique, the subtle increases in temperature and lower mass loss observed in the first degradation event of the prepared microcapsules, compared with the non-microencapsulated one, indicates an improvement in the thermal stability of the product after microencapsulation. Increase in thermal stability indicates that all properties of the beetroot and dairy drink mixture would be maintained even when exposed to high temperatures and that the bioactive compounds would be preserved during prolonged storage, ensuring their bioactivities and nutritional properties, even under variable temperature conditions [78].

EE determines how much of the active compound was effectively protected and retained within the microcapsule core and is considered an indirect indicator of the protective capacity, stability, functionality, and economic viability of the process. By indicating the proportion of the active compound that was encapsulated in relation to the initial quantity used, a high EE value shows that the process was successful and that there was little material loss during production [112]. Many bioactive compounds, such as phenolic compounds, organic acids, pigments like betalains, vitamins, essential oils, probiotics, and drugs, are easily degraded when exposed to light or oxygen, high water activity, and the physicochemical conditions or enzymes found in the gastrointestinal tract, resulting in poor bioavailability of compounds following oral administration and also leading to low bioavailability [112,113]. High EE can overcome this, but the efficiency of encapsulation depends on the capacity of the employed polysaccharides to hold betalains within their microparticles, which will probably take place through hydrogen bonding [112].

In the present study, the encapsulation efficiency was determined by considering the final content of betalains, as well as betacyanin and betaxanthins, retained in the microcapsule core. When just one encapsulating agent was used to build the microcapsule, cassava starch formulation showed the best encapsulation efficiency (>71%) of the original contents of betacyanins, betaxanthins and total betalains. Furthermore, when the mixture of maltodextrin and cassava starch was used to build the microcapsules, an improvement in EE was observed for betacyanins, betaxanthins and total betalains (>80%). Cassava starch is widely recognized as an excellent encapsulating agent due to its physicochemical, functional, and economic properties, making it highly competitive compared with other natural polymers. It exhibits high viscosity and paste clarity, forming stable and transparent solutions that favor the suspension and protection of bioactive compounds. Furthermore, it possesses good stability against freeze–thaw cycles, preventing phase separation and ensuring greater capsule durability. Its ability to form resistant and flexible films creates effective barriers against oxygen, light, and moisture, protecting sensitive molecules such as vitamins, antioxidants, essential oils, and probiotics from thermal or oxidative degradation, as well as reducing losses due to volatilization [114]. Being natural, biodegradable, non-toxic, and gluten-free, cassava starch is safe and widely accepted in food, pharmaceutical, and cosmetic formulations, ensuring biocompatibility and sustainability. Another highlight is its ability to promote the gradual and controlled release of encapsulated active ingredients, which increases functional efficacy and prolongs the action of core active compounds in different systems. From an economic standpoint, it is a low-cost and highly available input in tropical regions, especially in Latin America, Africa, and Asia, which makes the encapsulation process more accessible and sustainable. Although amylose retrogradation may occur over time, this challenge can be overcome by combining it with other encapsulating agents, such as maltodextrin or gum Arabic, further enhancing its properties [115,116]. These reinforce the strong potential of starch as an encapsulant, either alone or in combination, as the main contributor to the efficient retention of these bioactives.

Indeed, maltodextrin seems to enhance the efficiency of encapsulation, as recently demonstrated when gum Arabic mixed with maltodextrin, in different proportions, resulted in microparticles with greater pigment retention and better encapsulation efficiency compared with the use of single materials. This improvement was attributed to the individual functionality of the encapsulants, in which gum Arabic contributes to the emulsifying properties and film formation, while maltodextrin reduces hygroscopicity and favors product stability during drying [117]. Maltodextrin is considered one of the most efficient encapsulating agents when applied in freeze-drying processes, due to its physicochemical properties that favor the formation of stable and functional solid matrices. Being a polymer derived from the partial hydrolysis of starch, it exhibits high water solubility, low viscosity, and a neutral taste, characteristics that allow its use in food, pharmaceutical, and cosmetic systems without compromising sensory attributes. During freeze-drying, maltodextrin acts as a protective support, forming a solid network that surrounds and stabilizes sensitive bioactive compounds, such as vitamins, antioxidants, essential oils, and probiotics, reducing losses due to volatilization and preventing thermal or oxidative degradation processes. Furthermore, it contributes to decreasing the hygroscopicity of the resulting powders, ensuring greater fluidity, stability, and ease of handling. From an economic standpoint, maltodextrin is low-cost, widely available, and easy to process, making it a competitive alternative to other encapsulants, such as gum Arabic or modified starch. Although it shows less emulsifying power compared with some natural polymers, its combination with other agents can enhance results and broaden the range of applications [87,88].

In the present study, EE depends on the ability of polysaccharides to retain betalains in the microparticulate matrix, a process that occurs predominantly through intermolecular interactions, such as hydrogen bonds. The contribution of the present study consists in the development of a new functional dairy powdered product, in which the encapsulation is not restricted to the beetroot extract but extends to all constituents of the formulation. Cassava starch and maltodextrin showed an efficiency superior to 60% in retaining betalains, surpassing the non-microencapsulate formulation, where the absence of encapsulating agents possibly compromised the recovery of pigments. Similar results were observed in a study that evaluated the microencapsulation of beetroot soup using starch and maltodextrin through freeze-drying, with encapsulation efficiency greater than 50% for total betalains, betacyanins, and betaxanthins. Polysaccharides were effective in retaining these pigments within the matrix, promoting greater stability and reducing degradation during processing and storage. This efficiency was attributed to the intermolecular interactions between the betalains and the encapsulating agents, which form a protective barrier capable of minimizing pigment color losses. The results show the technological potential of polysaccharide microencapsulation for the development of new products [27].

Therefore, the high EE in powder formulations guarantee that betalains would be retained in the microcapsule nucleus, preserving the bioactive pigments and all beneficial compounds found in beetroots. The encapsulating matrix described herein was capable of preserving the outstanding antioxidant and anti-inflammatory properties of those beetroots pigments in order to protect organs such as the heart, liver, and kidneys by promoting the homeostasis of reactive oxygen species, reducing the inflammatory status of the organism; therefore, these propertied would be preserved thanks to the encapsulating matrix and would be able to regulate cellular functions [118].

Commercial shelf life established for the microencapsulated formulations reflects proper hygienic conditions during processing. However, development of new foods requires physicochemical evaluation and acceptability studies, where sensory analysis is an essential tool to understand the relationship between food and consumers, integrating knowledge from physiology, psychology, statistics, and food science. Therefore, sensory analysis is described as an essential tool for new-product development, quality control, and preference studies, because it involves not only the perception of sensory attributes—such as taste, aroma, texture, color, and appearance—but also the intensity, duration, and quality of sensations, as well as the acceptance or rejection that they may generate, influencing purchasing decisions [119]. In the present study, formulations microencapsulated in maltodextrin and the combination of maltodextrin and cassava starch presented the best scores regarding all attributes evaluated in the acceptance test and purchase intention, in particular the color attribute, which was the first parameter evaluated and got a value above 70%. This result indicates that maltodextrin seems to enhance the brightness of the formulation, as it was evaluated better than the dairy–beetroot powder drink without encapsulating agent and only encapsulated with cassava starch. Considering the aroma and texture, the evaluation was more balanced, but even so, the formulation microencapsulated in maltodextrin or that containing maltodextrin in its composition presented better results. This may indicate that the use of maltodextrin as an encapsulating agent contributed positively to product acceptance, possibly due to its technological properties, with emphasis on high solubility and ability to form stable matrices that protect volatile and bioactive compounds. It is likely that these characteristics may have favored the controlled release of aromatic compounds and the maintenance of a more pleasant texture to the palate, when compared with formulations encapsulated by using starch as the only encapsulating agent [87].

In addition, the taste and overall impression evaluations reinforce that maltodextrin microcapsules showed higher acceptance scores, suggesting that this encapsulating agent can attenuate undesirable residual notes of beets and improve sensory harmony. Considering the flavor attribute, it should be noted that the result might have been influenced by the inherent resistance of some consumers to the flavor of beets. In a study of beet and tomato juices, most raters indicated that they did not consume beet juice frequently, which may represent a reduced familiarity or a tendency to reject the characteristic flavor of this vegetable. Although juices with more intense colors are preferred visually, it does not necessarily translate into high flavor scores, suggesting that positive visual perception may not fully compensate for the sensory notes of beets [120].

All built microcapsules were able to preserve the bioactive compounds (betalains, NO_3_^-^ and saponins) in these innovative beverages, maintaining physicochemical stability in accordance with current legislation. However, the microencapsulated formulations with a maltodextrin and cassava combination showed the best results in terms of moisture, fiber, total sugars, and EE% of betacyanins, betaxanthins and total betalains. Despite having a high span index (less homogeneous), the dairy–beetroot powder drink formulated in a combination of maltodextrin and cassava presented low d(0.5) values, indicating the predominance of fine microparticles that favor its solubility. In addition, the dairy–beetroot powder drink microencapsulated in a combination of maltodextrin and cassava presented the best morphology of microcapsules, with spherical and vitreous particles.

It is important to mention that the present study has some limitations. Firstly, the stability assessment period of the formulation was relatively short, limited to only 30 days, which does not allow for extrapolating conclusions about its validity over longer time periods. Furthermore, despite a large sensory panel, the untrained individuals may or may not have introduced subjective variability in the sensory acceptance of the products. Another critical point is that the actual bioavailability of the beet active compounds was not tested in vivo, restricting the interpretation of potential physiological effects. Given these limitations, the need for well-designed clinical trials to confirm the bioavailability of these compounds could be evaluated by improving the endothelial function and hemodynamic parameters in randomized, controlled crossover studies after ingestion of the dairy–beetroot powder drink formulated in the cassava starch and maltodextrin combination.

## 5. Conclusions

The microencapsulated formulations proved to be an effective technological strategy to preserve betalains, NO_3_^-^ and saponins, ensuring preservation of the bioactive compounds in this innovative beverage and keeping physicochemical stability according to current legislation. Formulations with starch and a combination of starch and maltodextrin showed high encapsulation efficiency of betacyanins, betaxanthins and total betalains, whereas the formulation with maltodextrin and in the combination of starch and maltodextrin showed high solubility when reconstituted in ice water, favoring the retention of pigments and the balance of color, flavor and texture of the novel beetroot beverage. The morphology and thermogravimetric analyses confirmed the formation of stable microstructures against processing and elevated temperature, which expands the potential for technological application of the product as a durable food supplement, displaying antioxidant and vasoprotective effects. All samples obtained high sensory and acceptance values considering color attribute, demonstrating the positive influence of the polysaccharide on the perception of the final product. However, only formulations microencapsulated in maltodextrin and in the combination of maltodextrin and cassava starch received scores corresponding to “I liked it moderately” (score ≥ 7.0) for texture, taste attributes, and overall acceptability.

The dairy–beetroot powder drink formulations microencapsulated in cassava starch and maltodextrin are patented products according to the guidelines of the National Institute of Industrial Property (INPI), under number BR 10 20250219131. This beverage represents a promising alternative for the development of functional foods aimed at cardiovascular health and general well-being. It is recommended that complementary studies be developed concerning bioavailability, antioxidants and physiological response, including careful endothelial function and hemodynamic parameter evaluations in randomized controlled crossover trials following the highest rate formulation intake.

## Figures and Tables

**Table 1:** Proximate composition and bioactive compounds of freeze-dried dairy–beetroot powder drink microencapsulated formulations (100 g^−1^ fresh weight basis).

	Formulations			
	Dairy Base: Beetroot (2:1)	Dairy Base: Beetroot: Maltodextrin (2:1:1)	Dairy Base: Beetroot: Cassava Starch (2:1:1)	Dairy Base: Beetroot: 50% Maltodextrin and 50% Cassava Starch (2:1:1)
Energy (kcal)	373.59 ± 3.56 ^d^	473.66 ± 5.33 ^a^	459.70 ± 5.18 ^b^	468.84 ± 4.64 ^a,b^
Protein (g)	23.81 ± 2.86 ^a^	20.51 ± 1.92 ^a^	19.95 ± 1.88 ^a^	19.50 ± 1.97 ^a^
Lipid (g)	15.35 ± 0.77 ^a^	10.38 ± 1.14 ^b^	10.90 ± 0.96 ^b^	10.88 ± 0.63 ^b^
Carbohydrate (g)	35.05 ± 1.94 ^b^	74.55 ± 2.27 ^a^	70.45 ± 2.34 ^a^	73.23 ± 2.04 ^a^
Total dietary fiber (g)	4.81 ± 0.24 ^a^	3.84 ± 0.17 ^b^	3.92 ± 0.18 ^b^	3.68 ± 0.12 ^b^
Total sugars (g)	28.23 ± 2.57 ^a^	23.96 ± 1.66 ^b^	18.46 ± 1.78 ^c^	19.47 ± 1.85 ^c^
Fructose (g)	1.28 ± 0.11 ^a^	0.75 ± 0.19 ^b^	0.81 ± 0.10 ^b^	0.71 ± 0.16 ^b^
Sucrose (g)	15.07 ± 1.07 ^a^	8.28 ± 1.03 ^b^	8.36 ± 1.16 ^b^	8.66 ± 1.10 ^b^
Maltose (g)	ND	4.75 ± 0.11 ^a^	ND	1.22 ± 0.04 ^b^
Glucose (g)	4.94 ± 0.55 ^a^	3.26 ± 0.10 ^b^	2.27 ± 0.37 ^d^	2.16 ± 0.11 ^c^
Lactose (g)	6.43 ± 0.76 ^a^	6.37 ± 0.17 ^a^	6.49 ± 0.14 ^a^	6.22 ± 0.42 ^a^
Galactose (g)	0.51 + 0.08 ^a^	0.55 + 0.15 ^a^	0.53 + 0.11 ^a^	0.50 + 0.12 ^a^
NO_3_^-^ (mmol/mg)	15.97 ± 0.92/990.14 ^a^	10.01 ± 1.14/620.62 ^b^	10.91 ± 2.38/676.42 ^b^	10.07 ± 1.28/618.14 ^b^
NO_2_^-^ (mmol/mg)	0.51 ± 0.11/14.26 ^a^	0.28 ± 0.07/12.88 ^b^	0.21 ± 0.09/9.66 ^b^	0.22 ± 0.08/10.12 ^b^
Saponins (mg)	3755 ± 88.48 ^a^	2093 ± 73.04 ^b^	2115 ± 91.36 ^b^	2015 ± 33.16 ^b^
Moisture (%)	16.20 ± 1.12 ^a^	10.35 ± 0.74 ^b^	10.24 ± 0.92 ^b^	10.14 ± 0.83 ^b^
Ashes (%)	5.59 ± 0.16 ^a^	3.63 ± 0.56 ^b^	3.74 ± 0.45 ^b^	3.19 ± 0.22 ^b^

Values expressed as means ± standard deviations (n = 3). Different letters within the same line indicate significant differences between samples (*p* < 0.05). ND, not determined; NO_2_^-^, nitrite; NO_3_^-^, nitrate.

**Table 2:** Particle size distribution of freeze-dried dairy–beetroot powder drink microparticles.

Microparticle Diameters (μm)				
Formulations	d (0.1)	d (0.5)	d (0.9)	*Span*
Dairy base: beetroot: maltodextrin (2:1:1)	4.13 ± 0.77 ^c^	15.58 ± 0.86 ^c^	166.82 ± 2.74 ^d^	10.45 ± 0.46 ^b^
Dairy base: beetroot: cassava starch (2:1:1)	9.78 ± 0.98 ^b^	76.45 ± 0.93 ^b^	395.13 ± 2.55 ^b^	5.13 ± 0.77 ^c^
Dairy base: beetroot: 50% maltodextrin and 50% cassava starch (2:1:1)	4.82 ± 0.64 ^c^	16.44 ± 1.05 ^c^	316.01 ± 1.86 ^c^	18.94 ± 0.56 ^a^
Dairy base: beetroot (2:1; no encapsulating agent)	20.39 ± 0.82 ^a^	163.17 ± 2.15 ^a^	458.37 ± 3.37 ª	2.68 ± 0.81 ^d^

Values are expressed as means ± SDs (n = 3). Different letters in the same column indicate significant differences between samples (*p* < 0.05). Results are displayed as d (0.1), d (0.5) and d (0.9), corresponding to the maximum size (in μm) from 10%, 50% or 90% of microparticles.

**Table 3:** Experimental design, composition, yield and physicochemical properties of freeze-dried dairy–beetroot powder drink microparticles.

	Formulations			
	Dairy Base: Beetroot: Maltodextrin (2:1:1)	Dairy Base: Beetroot: Cassava Starch (2:1:1)	Dairy Base: Beetroot: 50% Maltodextrin and 50% Cassava Starch (2:1:1)	Dairy Base: Beetroot (2:1; No Encapsulating Agent)
Dairy base (g)	51	51	51	51
Beetroots (g)	12.5	12.5	12.5	12.5
Starch (g)	-	12.5	6.25	-
Maltodextrin (g)	12.5	-	6.25	-
Total Solids (%)	76	76	76	63
Yield (%)	66.36 ± 2.22 ^a^	68.02 ± 2.52 ^a^	64.74 ± 2.13 ^a^	21.41 ± 2.19 ^b^
*a_w_*	0.30 ± 0.02 ^b^	0.21 ± 0.03 ^a^	0.22 ± 0.02 ^a^	0.37 ± 0.01 ^c^
Water solubility index (%)	91.02 ± 2.71 ^b^	87.27 ± 2.45 ^b^	97.86 ± 2.30 ^a^	55.12 ± 0.42 ^c^
Water absorption index (%)	72.42 ± 1.82 ^d^	134.18 ± 1.53 ^b^	113.37 ± 1.48 ^c^	212.04 ± 2.37 ^a^
pH	4.85 ± 0.18 ^a^	4.72 ± 0.09 ^a^	4.76 ± 0.12 ^a^	4.66 ± 0.07 ^a^

Values are expressed as means ± SDs. Different letters in the same line indicate statistically significant differences between samples (*p* < 0.05). *a_w_*, water activity.

**Table 4:** Encapsulation efficiency of freeze-dried dairy–beetroot powder drink microparticles, using betacyanins, betaxanthins and total betalains as indicators for compound retention in microparticle core.

Formulations	Contents (mg·g^−1^)			EE (%)		
	Betacyanins	Betaxanthins	Total Betalains	Betacyanins	Betaxanthins	Total Betalains
Dairy base: beetroot (2:1; no encapsulating agent)	0.176 ± 0.06 ^c^	0.246 ± 0.08 ^b^	0.334 ± 0.05 ^c^	-	-	-
Dairy base: beetroot: maltodextrin (2:1:1)	0.279 ± 0.07 ^b^	0.248 ± 0.04 ^b^	0.427 ± 0.04 ^b^	64.29 ± 0.67 ^c^	62.21 ± 0.15 ^c^	63.55 ± 0.41 ^c^
Dairy base: beetroot: cassava starch (2:1:1)	0.411 ± 0.06 ^a^	0.349 ± 0.06 ^a^	0.760 ± 0.04 ^a^	71.28 ± 0.42 ^b^	72.53 ± 0.27 ^b^	74.41 ± 0.33 ^b^
Dairy base: beetroot: 50% maltodextrin and 50% cassava starch (2:1:1)	0.404 ± 0.09 ^a^	0.353 ± 0.05 ^a^	0.757 ± 0.11 ^a^	80.12 ± 0.76 ^a^	78.76 ± 0.36 ^a^	81.75 ± 0.86 ^a^

Values are expressed as means ± SDs. Different letters in the same column indicate differences between samples (*p* < 0.05). The quantification and efficiency of the dairy base: beetroot (2:1; no encapsulating agent) sample were not determined. EE, encapsulation efficiency.

**Table 5:** Microbiological analyses of dairy–beetroot powder drink microparticles according to guidelines from Brazilian legislation *.

Microbiological Analyses	Dairy Base: Beetroot (2:1; No Encapsulating Agent)	Dairy Base: Beetroot: Maltodextrin (2:1:1)	Dairy Base: Beetroot: Cassava Starch (2:1:1)	Dairy Base: Beetroot: 50% Maltodextrin and 50% Cassava Starch (2:1:1)	Limits Established by Brazilian Current Legislation *
Analyses at 0 day of storage					
*Staphylococcus* coagulase-positive (CFU·g^−1^)	ND	ND	ND	ND	-
Coliforms at 45 °C (MPN·g^−1^)	ND	ND	ND	ND	-
Total coliforms (MPN·g^−1^)	Absent	Absent	Absent	Absent	Absent
*Salmonella* spp. (25 g)	Absent	Absent	Absent	Absent	Absent
*Bacillus cereus* (CFU·g^−1^)	ND	ND	ND	ND	-
*Esherichia coli* (MPN·g^−1^)	ND	ND	ND	ND	5 × 10^1^ MPN·g^−1^
Molds, yeasts and fungi (CFU·g^−1^)	2.16 × 10^1^	1.80 × 10^1^	1.71 × 10^1^	1.13 × 10^1^	5 × 10^3^ CFU·g^−1^
Analyses on 14th day of storage					
*Staphylococcus* coagulase-positive (CFU·g^−1^)	ND	ND	ND	ND	-
Coliforms at 45 °C (MPN·g^−1^)	ND	ND	ND	ND	-
Total coliforms (MPN·g^−1^)	Absent	Absent	Absent	Absent	Absent
*Salmonella* spp. (25 g)	Absent	Absent	Absent	Absent	Absent
*Bacillus cereus* (CFU·g^−1^)	ND	ND	ND	ND	-
*Esherichia coli* (MPN·g^−1^)	ND	ND	ND	ND	5 × 10^1^ MPN·g^−1^
Molds, yeasts and fungi (CFU·g^−1^)	2.47 × 10^1^	1.98 × 10^1^	1.45 × 10^1^	1.01 × 10^1^	5 × 10^3^ CFU·g^−1^
Analyses on 30th day of storage					
*Staphylococcus* coagulase-positive (CFU·g^−1^)	ND	ND	ND	ND	-
Coliforms at 45 °C (MPN·g^−1^)	ND	ND	ND	ND	-
Total coliforms (MPN·g^−1^)	Absent	Absent	Absent	Absent	Absent
*Salmonella* spp. (25 g)	Absent	Absent	Absent	Absent	Absent
*Bacillus cereus* (CFU·g^−1^)	ND	ND	ND	ND	-
*Esherichia coli* (MPN·g^−1^)	ND	ND	ND	ND	5 × 10
Molds, yeasts and fungi (CFU·g^−1^)	4.10 × 10^1^	2.34 × 10^1^	2.71 × 10^1^	2.47 × 10^1^	5 × 10^3^

CFU·g^−1^, colony-forming units per gram; MPN∙g^−1^, most likely number per gram of powdered beetroot soup. * Limits established by current Brazilian legislation Resolution No. 331 of 23 December 2019.

**Table 6:** Sensorial evaluation and purchase intention of dairy–beetroot powder drink.

Dairy–Beetroot Powder Drink				
Sensory Attributes	Dairy Base: Beetroot (2:1; No Encapsulating Agent)	Dairy Base: Beetroot: Maltodextrin (2:1:1)	Dairy Base: Beetroot: Cassava Starch (2:1:1)	Dairy Base: Beetroot: 50% Maltodextrin and 50% Cassava Starch (2:1:1)
Color	7.16 ± 0.44 ^a^	7.90 ± 0.30 ^a^	7.53 ± 0.54 ^a^	7.71 ± 0.59 ^a^
Aroma	6.33 ± 0.44 ^a^	6.83 ± 0.45 ^a^	6.09 ± 0.60 ^a^	6.66 ± 0.54 ^a^
Texture	7.06 ± 0.16 ^b^	7.84 ± 0.27 ^a^	6.16 ± 0.34 ^c^	7.96 ± 0.16 ^a^
Taste	6.93 ± 0.12 ^b^	7.80 ± 0.14 ^a^	6.40 ± 0.17 ^c^	7.43 ± 0.14 ^a^
Overall acceptability	6.87 ± 0.29 ^b^	7.89 ± 0.50 ^a^	5.94 ± 0.32 ^c^	7.77 ± 0.41 ^a^
Purchase intention	3.94 ± 0.09 ^b^	4.43 ± 0.14 ^a^	3.48 ± 0.11 ^c^	4.28 ± 0.21 ^a^

Values are expressed as means ± SDs (n = 3). Different letters within the same line denote significant differences (one-way ANOVA and Bonferroni post-test; *p* < 0.001). Acceptance attributes of aroma, color, taste and overall acceptability were assessed by applying a structured 9-point hedonic scale ranging from 1 = disliked it extremely to 9 = liked it very much. Purchase intention was evaluated using a structured 5-point hedonic scale ranging from 1 = would certainly not buy it to 5 = would certainly buy it.

## Data Availability

The original contributions presented in this study are included in the article. Further inquiries can be directed to the corresponding author.
